# Identification of Potential Proteins Interacting with α-Galactosidase A to Analyze the Pathogenesis of Fabry Disease

**DOI:** 10.3390/ijms27125437

**Published:** 2026-06-16

**Authors:** Elise Raphaela Menke, Jürgen Eirich, Iris Finkemeier, Malte Lenders, Eva Brand

**Affiliations:** 1 Internal Medicine D (Nephrology, Hypertension and Rheumatology), and Interdisciplinary Fabry Center (IFAZ), University Hospital Muenster, Albert-Schweitzer-Campus 1, 48149 Muenster, Germany; eliseraphaela.menke@ukmuenster.de (E.R.M.);; 2 Institute of Plant Biology and Biotechnology, University of Muenster, 48143 Muenster, Germany

**Keywords:** Fabry disease, α-galactosidase A, TurboID, proteomics

## Abstract

The lysosomal enzyme α-galactosidase A (AGAL) degrades globotriaosylceramide (Gb_3_). While this enzymatic function in lysosomal metabolism is well characterized, interaction partners and alternative functions are unknown. This study aims to identify new potential AGAL-interacting proteins. AGAL was fused to the mutated biotin ligase BirA from *E. coli* (TurboID). Expression of the fusion protein was confirmed by Western blot and immunofluorescence, while enzymatic activity was verified by functional assays. In three experimental settings (AGAL wild-type (WT), AGAL missense variant (p.N215S), and the control cell line), TurboID-biotinylated proximal proteins were enriched by streptavidin pull-down and analyzed by mass spectrometry. Gene Ontology (GO) terms were subsequently evaluated to characterize biological functions and localizations of the identified proteins. Selected candidates were co-immunoprecipitated with AGAL to confirm direct interactions. The AGAL-TurboID fusion protein was successfully expressed in AB8/13 podocytes. Immunofluorescence and enzyme activity assays confirmed the presence and functionality of the fusion protein. Subsequent functional analysis (GO term analysis) showed enrichment of driver terms, including *extracellular matrix organization* (ECM), *multicellular organism development*, and *protein metabolic process*, in the biological process category. The identified top-hit proteins were predominantly involved in the organization of ECM, cell proliferation and cytokinesis, unfolded protein response during endoplasmic reticulum stress, and protein ubiquitination. Co-immunoprecipitation confirmed the interaction between AGAL and the candidate Galectin-3-binding protein (Gal-3BP). Our results suggest that AGAL may play a role in other pathways and/or the ECM organization beyond its lysosomal function. The confirmed interaction with Gal-3BP can now be functionally investigated in further studies.

## 1. Introduction

Fabry disease (FD) is an X-linked lysosomal storage disorder caused by mutations in the *GLA* gene, which encodes the enzyme α-galactosidase A (AGAL).

AGAL is a lysosomal hydrolase mainly responsible for the breakdown of its substrate globotriaosylceramide (Gb_3_) and related glycolipids, maintaining cellular lipid homeostasis and thus preventing toxic substrate accumulation. X-ray analysis of the enzyme revealed a homodimer with two identical monomers [1]. A deficiency in AGAL activity leads to the accumulation of lysosomal substrates with terminal α-linked galactose, in particular, globotriaosylceramide (Gb_3_) [2]. This can cause the characteristic symptoms of FD, including ischemic stroke, heart failure, renal failure, and small-fiber neuropathy [3]. In the kidney, Gb_3_ accumulation in podocytes plays a crucial role, leading to foot process effacement with albuminuria and reduced glomerular filtration [4]. While the enzymatic function of AGAL in lysosomal metabolism is well characterized, other interaction partners and possible alternative functions are still unknown. Various proteomic analyses have been performed to investigate the effects of AGAL deficiency on cellular processes. These analyses showed that the composition of plasma proteins (involved, e.g., in pH regulation, glutathione metabolism, inflammation, and extracellular matrix (ECM) homeostasis) is altered in patients with FD compared with the controls [5]. The observed changes affect the cytokine-mediated pathway, ECM, and lysosomal proteome [5]. In addition, high-throughput omics technologies (including LC-MS, CE-MS, microarray, etc.) have been used to compare treatment-naïve patients with enzyme replacement therapy (ERT)-treated patients with FD [6], indicating differences in ECM remodeling, acute inflammatory response, regulation of wound healing, cellular response to reactive oxygen species, and regulation of peptidase activity [6]. An analysis of *GLA*-knockout podocytes identified an altered protein composition affecting endocytosis, thermogenesis, lysosomal trafficking, lysosomal function, actin skeleton regulation, cell signaling, and tight junctions [7]. Importantly, these effects could only be partially corrected by rescue experiments. Moreover, observed changes in protein composition have been further described in endothelial, epithelial, and patient-derived urinary fibroblasts, indicating cross-cell-type effects [7].

These studies show the effects of AGAL deficiency beyond Gb_3_ deposition and more complex cellular dysfunction. However, it is unclear to what extent the observed changes are a direct consequence of AGAL deficiency, a possible misfolding associated with certain mutations, or indirect consequences of Gb_3_ deposition.

In addition to an interaction with itself (as a homodimer), the substrate and general proteins, including molecular chaperones, only two AGAL-interacting proteins have been reported: the cation-independent mannose-6-phosphate receptor (CI-MPR) and saposin B. While the CI-MPR is responsible for proper intracellular trafficking of AGAL from the endoplasmic reticulum to the lysosomes, saposin B has been reported as a potential activator protein for AGAL, enhancing its activity in an artificial detergent-free liposomal system [8,9].

The aim of this study is to identify potential new interaction partners of AGAL that are associated with its cellular function and may contribute to the pathogenesis of FD.

## 2. Results

### 2.1. Vector Construction and Functionality Testing

**Figure 1:**
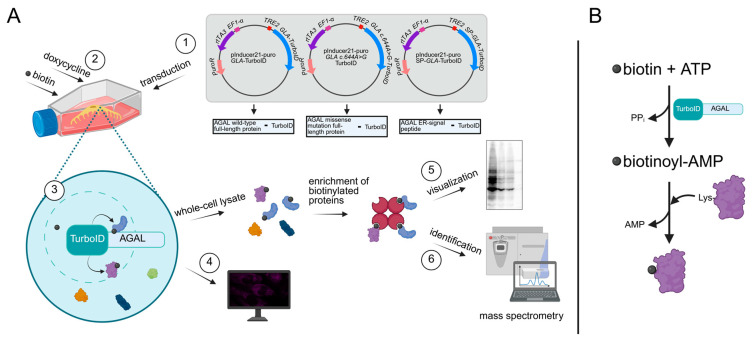
**A schematic overview of TurboID-based proximity labeling in AB8/13 cells.** (**A**) ① One of the shown constructs was transduced into AB8/13 wild-type cells using a lentiviral approach. The constructs encode a fusion protein consisting of the modified biotin ligase TurboID and either AGAL-WT, the AGAL-p.N215S mutation, or the ER-signal peptide sequence of AGAL (AGAL-SP). ② Following successful transduction, expression of the fusion protein was induced by doxycycline, and biotin was then added to initiate proximity labeling. ③ Within the cell, proteins in close proximity to the fusion protein were labeled (~10 to 20 nm). ④ The expression of the fusion protein and the biotinylation of proximal proteins were then visualized by immunofluorescence. ⑤ The biotinylated proteins were isolated from the whole-cell lysate using streptavidin-conjugated Dynabeads and were then detected using a Western blot. ⑥ The biotinylated proteins were identified by mass spectrometry. Proteins significantly enriched in AGAL-WT and/or AGAL-p.N215S-TurboID samples compared with the AGAL-SP-TurboID control were used for GO term analysis. (**B**) A schematic representation of the TurboID-catalyzed reaction. The reaction is initiated by the formation of the reactive intermediate biotinoyl-5′-adenylate (biotinoyl-AMP) with the simultaneous release of pyrophosphate (PP_i_). Subsequent to this, biotinoyl-AMP reacts with primary amino groups of lysine residues. With the elimination of AMP, the protein is covalently biotinylated.

**Figure 2:**
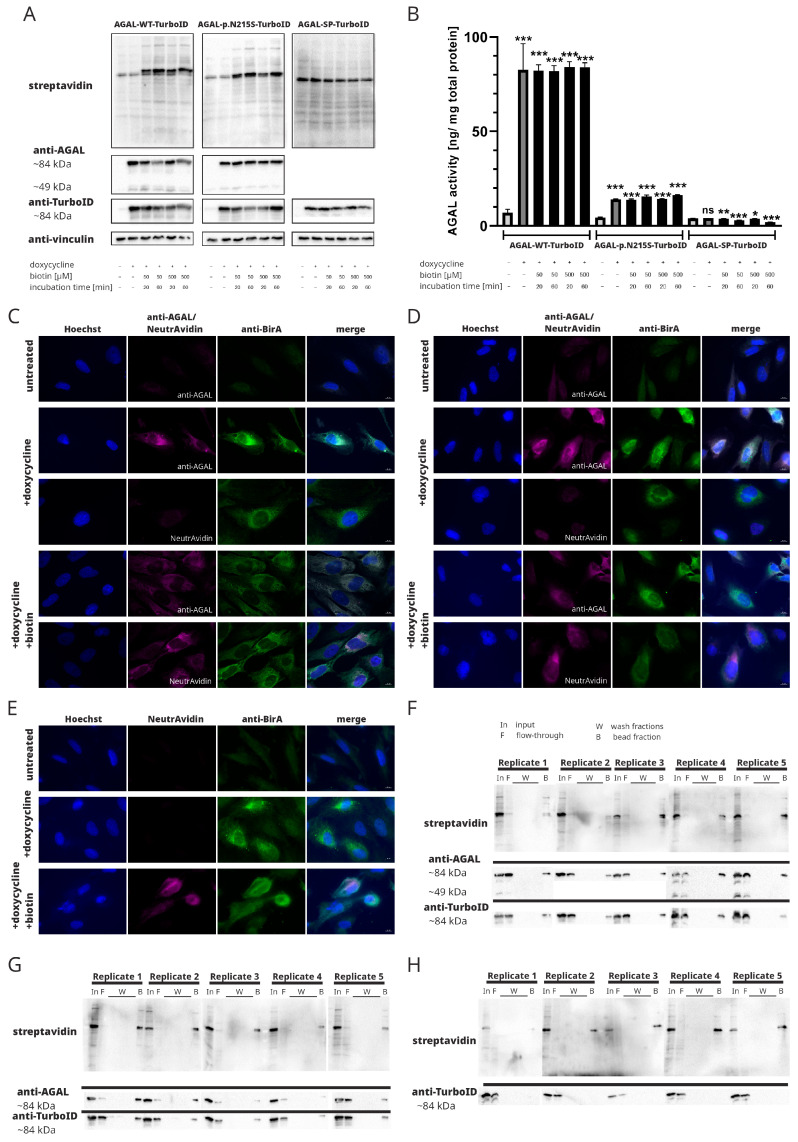
**Validation of the expression and functionality of the TurboID-fusion proteins.** (**A**) A Western blot analysis of biotinylated proteins and TurboID-fusion proteins in the three cell lines (AGAL-WT-, AGAL-p.N215S-, and AGAL-SP-TurboID), detected with anti-AGAL and anti-BirA antibodies. Confirmation of the doxycycline-dependent inducibility of the fusion proteins and increasing biotinylation with longer incubation time and higher biotin concentrations in non-control samples. Based on these results, an incubation of 20 min with 50 µM biotin was chosen for further experiments. (**B**) AGAL enzyme activities under the conditions shown in (**A**). The increased AGAL activity after doxycycline addition confirmed the inducible expression of the fusion proteins; the activity in the absence of doxycycline corresponds to the endogenous AGAL activity. (**C**–**E**) Immunofluorescence analysis of the three cell lines (AGAL-WT- (**C**), AGAL-p.N215S (**D**), and AGAL-SP-TurboID (**E**)) showed a ubiquitous localization of the fusion proteins (stained with anti-AGAL and anti-BirA (TurboID)) and the biotinylated proteins (labeled with NeutrAvidin). Untreated cells showed only background signal in all three conditions. The length of the scale bar is 10 µm. (**F**–**H**) A Western blot analysis of the samples (AGAL-WT- (**F**), AGAL-p.N215S (**G**), and AGAL-SP-TurboID (**H**)) used for mass spectrometry (five biological replicates per cell line). Shown are the biotinylated proteins before and after streptavidin pull-down (IN, B), as well as the corresponding flow-through and wash fractions. The biotinylated proteins were efficiently isolated using streptavidin beads. Fusion proteins were primarily detected in the input, flow-through, and bead fractions with anti-AGAL (**F**–**G**) and/or anti-BirA (**F**–**H**). IN: input, F: flow-through fraction, W: wash fraction, B: bead. ns: non significant. * *p* < 0.05, ** *p* < 0.01, *** *p* < 0.001.

Three different vectors were constructed for TurboID-based proximity labeling. The constructs contained the cDNA for the modified biotin ligase TurboID and either the human *GLA* WT cDNA sequence, the *GLA* cDNA sequence with the mutation c.644A > G (p.N215S), or only the N-terminal ER-signal peptide sequence of the *GLA* cDNA (Figure 1A). Expression of the fusion proteins was verified by Western blot analysis (Figure 2A). The full-length WT fusion protein was expected and visible at a height of approximately 84 kDa. To verify that TurboID can biotinylate other proteins as a fusion protein with AGAL, biotinylated proteins were detected in the cell lysate. A time- and concentration-dependent increase in the biotinylation of proteins was observed in AGAL-WT- and AGAL-p.N215S-TurboID samples (Figure 2A). To minimize nonspecific biotinylation and background signals, the shortest possible incubation time and a low biotin concentration (50 µM biotin, 20 min) were chosen for subsequent experiments. AGAL activity assays demonstrated the functionality of fusion proteins (Figure 2B). The enzyme activity of the sample without doxycycline addition corresponded to the endogenous activity. AGAL-WT-TurboID showed the highest enzyme activity, followed by AGAL-p.N215S-TurboID. As expected, the AGAL-SP-TurboID control showed no additional activity to the endogenous AGAL activity. Of note, compared with uninduced samples, the amount of overexpressed AGAL-WT-TurboID was only about 12-fold increased, demonstrating a very moderate level of overexpression (Figure 2B). To further ensure correct expression, the cellular localization of the fusion proteins was analyzed by immunofluorescence (Figure 2C–E). Unstimulated cells showed only background signal in all three cell lines. The addition of doxycycline induced the expression of the fusion protein, and a ubiquitous localization was visible in all three cell lines. The localization was visualized with anti-BirA (all) and anti-AGAL (AGAL-WT- and AGAL-p.N215S-TurboID). Biotinylated proteins (detection with NeutrAvidin) became only visible after the addition of biotin.

### 2.2. Isolation of Biotinylated Proteins

For the isolation of potential interacting proteins, the biotinylated proteins were isolated from the whole-cell lysate using streptavidin-coated Dynabeads (Figure 1A). Pull-downs of the fusion proteins and biotinylated proteins were analyzed in all fractions by Western blot analysis (Figure 2F–H). The fusion proteins were detected using anti-AGAL and anti-BirA antibodies, both of which detected matching protein bands (Figure 2F–G). Endogenous AGAL (approx. 49 kDa) was partially detectable in the AGAL-WT-TurboID sample but was not visible in other samples due to the strong signal from the overexpressed fusion protein. The fusion protein was detected primarily in the input, flow-through, and bead fractions. Since the biotinylation of proteins takes place in close proximity, a high degree of self-biotinylation was also expected. Detection in the flow-through fraction suggests that not all of the biotinylated proteins were isolated by the beads. However, the majority of the biotinylated proteins (visualized with streptavidin) were detected in the input and bead fractions.

### 2.3. Identification of Potential Interacting Proteins Using Mass Spectrometry

**Figure 3:**
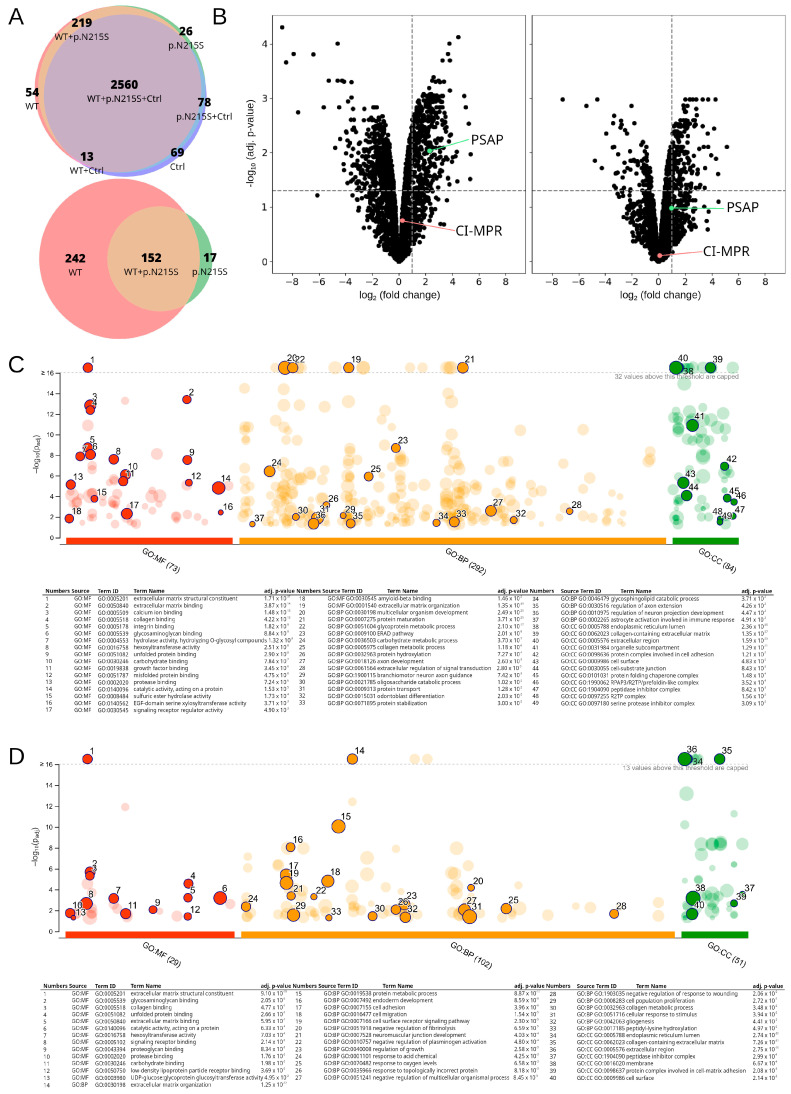
**Differently enriched proteins in AGAL-WT-TurboID and AGAL-p.N215S-TurboID and their functional annotation by GO analysis.** (**A**) The total number of proteins identified in the AGAL-WT-, AGAL-p.N215S-, and AGAL-SP-TurboID samples, based on five biological replicates each (in total, 3019). The Venn diagram below shows potential interaction partners in AGAL-WT- and AGAL-p.N215S-TurboID samples. Only proteins significantly enriched compared with the AGAL-SP-TurboID control (fold change > 1; adjusted *p*-value ≤ 0.05) are shown (in total, 411). (**B**) A volcano plot of the enriched proteins in the AGAL-WT- (left) and AGAL-p.N215S-TurboID (right) samples compared with the AGAL-SP-TurboID control. Significant proteins (fold change > 1; adjusted *p*-value ≤ 0.05) are located in the upper right quadrants. (**C**) GO analysis of the proteins enriched in the AGAL-WT-TurboID samples, analyzed using g:profiler. Functional classification is performed in the *molecular function* (MF), *biological process* (BP), and *cellular compartment* (CC, subcellular location). The circle size represents the term size; the GO terms that are closely related (GO subtree) are arranged closer to each other. Highlighted “driver terms” were identified using a two-step algorithm. (**D**) GO analysis of the proteins enriched in the AGAL-p.N215S-TurboID samples. CI-MPR: cation-independent mannose 6-phosphate receptor, and PSAP: prosaposin.

After excluding common contamination proteins (including albumin, casein, lysozyme C, and trypsin), a total of 3019 protein groups were identified in the AGAL-WT-, AGAL-p.N215S-, and AGAL-SP-TurboID samples (Figure 3A). Proteins that were present in at least three of the five biological replicates were included in further analyses. The largest proportion (2560 proteins) was detectable and common in all samples. A subset of 219 proteins were found in both the AGAL-WT- and AGAL-p.N215S-TurboID samples.

To identify potential interacting proteins, only proteins that were significantly enriched compared with the control sample (threshold fold change > 1 and adjusted *p*-value ≤ 0.05) were further included. Of these 411 identified proteins, 152 proteins were significantly enriched in both AGAL-WT- and AGAL-p.N215S-TurboID samples (Figure 3A). The majority were significantly enriched exclusively in the AGAL-WT-TurboID samples. The known interaction partner of AGAL, the CI-MPR, was also found in the samples (marked in Figure 3B). This receptor mediates the transport of endogenous AGAL from the *trans*-Golgi network to endosomes, and finally to lysosomes, as well as the cellular uptake of ERT [10,11]. A second suggested interaction partner, saposin B, was confirmed in the AGAL-WT-TurboID samples by an enrichment of PSAP (adj. *p*-value: 0.0091, log_2_ fold change: 2.30, and adj. *p*-value: 0.1052; log_2_ fold change: 0.97; Figure 3B).

### 2.4. Gene Ontology Analysis of Identified Proteins

For an initial functional assessment of the enriched proteins, the significant Gene Ontology (GO) terms of the samples were determined, which are divided into three categories: *molecular function* (MF), *biological process* (BP), and *cellular compartment* (CC) (Figure 3C,D). In Figure 3C,D, the respective driver terms (further filtering of the GO terms) are highlighted. For the AGAL-WT-TurboID samples, a total of 73 MF terms (19 driver terms), 292 BP terms (18 driver terms), and 84 CC terms (12 driver terms) were identified (Figure 3C). MF terms include *ECM binding* (including collagen, integrin, and glycosaminoglycan binding), *catalytic activity* (including hydrolase, transferase, and protease), and *unfolded/misfolded protein binding* (including heat shock protein, chaperone, and folding sensor protein). The number of proteins per BP term relative to −log_10_ (adj. *p*-value) is shown in Supplementary Figure S1A. The term *multicellular organism development* had the most assignments with 182 proteins, followed by *ECM organization* (60) and *protein maturation* (58). The CC terms primarily include the ER lumen, extracellular region, cell surface, or belong to the R2TP complex or chaperone complex (Figure 3C). For the AGAL-p.N215S-TurboID samples, 29 MF terms (13 driver terms), 102 BP terms (20 driver terms), and 51 CC terms (7 driver terms) were identified (Figure 3D). MF terms primarily include *ECM binding* (including collagen and glycosaminoglycan binding), *catalytic activity* (including hydrolase and transferase), *receptor binding* (including low-density lipoprotein), and *unfolded protein binding* (including heat shock protein, chaperone, and folding sensor protein). The BP term analysis shows that 84 proteins were assigned to the term *cellular response to stimulus*, followed by the *protein metabolic process* (81) and the *cell surface receptor signaling pathway* (50) (Supplementary Figure S1B). The CC terms mainly include ER lumen, extracellular region, cellular membrane, cell surface, and peptidase inhibitor complex (Figure 3D).

One of the central GO terms in both compared groups was *ECM organization* (Supplementary Figures S1 and S2A). This term primarily refers to numerous collagens, collagen-associated proteins, TGF-beta and TGF-beta-associated proteins, laminins, and membrane proteins found in our samples. These proteins play an important role in cell adhesion, migration, polarization, and structural stability [12,13].

### 2.5. Analysis of Top Hits

For further analysis, the top hits were determined based on a combined score of fold change and adjusted *p*-value (Supplementary Figure S3, Supplementary Tables S2 and S3). Again, there was a clear enrichment of proteins involved in the organization of the ECM for both the AGAL-WT- and AGAL-p.N215S-TurboID samples, including several collagens and ECM remodeling proteins (transforming growth factor beta-2 proprotein and SPARC-related modular calcium-binding protein 1). The top hits for the AGAL-WT-TurboID samples also included the ECM proteins laminin subunit beta-2, Galectin-3-binding protein (Gal-3BP), and latent-transforming growth factor beta-binding protein 3, while the AGAL-p.N215S-TurboID samples increasingly contained collagen processing protein (protein-lysine 6-oxidase) and integrin (integrin alpha-11).

In addition, other proteins involved in processes such as cell proliferation and cytokinesis (nuclear migration protein nudC), the unfolded protein response during endoplasmic reticulum (ER) stress (DnaJ homolog subfamily C member 3), protein ubiquitination (E3 ubiquitin-protein ligase RNF216), and chaperonin-containing T-complex (T-complex protein 1 subunit theta) and R2TP complex (RNA polymerase II-associated protein 3) were detected in both conditions (AGAL-WT- and AGAL-p.N215S-TurboID samples).

The AGAL-WT-TurboID samples also included proteins with functions related to receptor binding (chaperone Alpha-2-macroglobulin receptor-associated protein), NF-κB (inhibitor of nuclear factor kappa-B kinase-interacting protein), disulfide bond formation (ERO1 like protein beta), and heparan sulfate modification (extracellular sulfatase Sulf-2). The AGAL-p.N215S-TurboID samples showed an increased abundance of proteins involved in proliferation (Chondroitin sulfate proteoglycan 4), as well as an RNA helicase (Probable ATP-dependent RNA helicase DDX10).

### 2.6. Differences in Protein Enrichments Between AGAL-WT and AGAL-p.N215S

Subsequently, a further comparison between WT and the variants was performed to identify differentially enriched candidates. The comparison was based on a combined score of fold change and adjusted *p*-value (Supplementary Figure S2B). Proteins with high positive scores were significantly enriched compared with the control. Deviations from the diagonal indicate proteins that were not only significantly enriched compared with the control, but also occurred in different amounts between the two conditions. For example, the protein RNA polymerase II-associated protein 3 (RPAP3) was significantly enriched in both conditions, but more pronounced in the AGAL-WT TurboID sample.

In addition, other proteins involved in various relevant processes were enriched, and more were pronounced in the AGAL-WT TurboID sample. FAD-dependent oxidoreductase domain-containing protein 1 (FOXRED1) is essential for the assembly of mitochondrial respiratory chain complex I and has oxidoreductase activity [14]. Cerebellin 2 Precursor (CBLN2) is involved in the formation and function of synapses, with low expression in kidney, liver, eye, and gastrointestinal tract [15,16]. BAG2 acts as a co-chaperone of hsp70 and hsc70, promoting the release of ADP and substrates [17,18]. Vitamin K-dependent protein S is a glycoprotein regulating coagulation; it is also involved in inflammatory processes [19]. Testican-1 (SPOCK1) is involved in cell–matrix interaction [20,21]. A protein more enriched in the AGAL-p.N215S sample is the RNA helicase DDX10, which is associated with RNA processing and inflammatory signaling pathways [22].

### 2.7. Co-Immunoprecipitation

**Figure 4:**
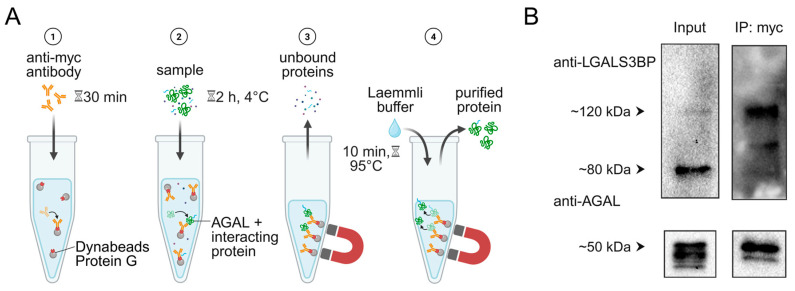
**Verification of AGAL-interacting protein Gal-3BP by co-IP.** (**A**) A schematic illustration of a co-IP experiment. Dynabeads Protein G were incubated with an anti-myc antibody (①), followed by the addition of lysates from transfected AB8/13-cells (②). After removal of unbound proteins (③), bound protein complexes were eluted and subsequently analyzed using Western blot (④). (**B**) Western blot analysis with anti-LGALS3BP and anti-AGAL antibodies (*n* = 3). The controls (beads only, as well as a control with beads and anti-myc antibody, but without lysate) show no signals at the respective heights.

To verify the potential direct interactions with AGAL, proteins from the top hits were selected for further co-immunoprecipitation (co-IP) experiments. The co-IP was performed using Protein G Dynabeads. An interaction with the Gal-3BP was detected (Figure 4). The presence of an additional visible band in the Western blot analysis may indicate an isoform of the protein. No interaction could be confirmed in the co-IP experiments with the additional four candidates, since no bands were detected in the Western blot analysis. These candidates were low-density lipoprotein receptor-related protein-associated protein 1, DnaJ homolog subfamily C member 1, RNA Polymerase II Associated Protein 3, and E3 ubiquitin-protein ligase RNF216.

## 3. Discussion

The lysosomal function of AGAL, which includes the hydrolysis of terminal α-galactosyl residues of glycolipids, is well understood. However, potential interaction partners are largely unknown.

This study aimed to identify potential protein interaction partners of AGAL. Our main results demonstrate that: (1) TurboID-based proximity labeling appears to be a suitable method for identifying proximal AGAL proteins in vivo; (2) after applying predefined significance criteria, 411 potential AGAL-interacting proteins were identified; (3) identified proteins belonged predominantly to *ECM organization*, *multicellular organism development*, and *protein metabolic processes*; and (4) co-IP confirmed Gal-3BP as an interaction partner of AGAL.

### 3.1. Potential AGAL Interaction Partners and Their Potential Role in FD Pathogenesis

Following translation into the ER and attachment of three carbohydrate chains (N-glycosylation, positions p.N139, p.N192, p.N215), AGAL undergoes post-translational modification in the Golgi apparatus [11,23]. During this process, the mannose residues of the carbohydrate chains are phosphorylated. AGAL is then transported to the lysosomes via the CI-MPR [11,23]. A small amount is misorted in the *trans*-Golgi Network and secreted [10,11]. AGAL can also be taken up into the cell via the membrane-bound CI-MPR. Therefore, it seems possible that AGAL interacts with other proteins during intracellular transport or in the extracellular space and may have additional functions.

Interestingly, a significant enrichment of saposin B was only observed in the AGAL-WT TurboID samples and not for AGAL-p.N215S. This could be explained by the fact that the p.N215S variant results in insufficient glycosylation, causing a retention of the protein in the endoplasmic reticulum [24]. As a consequence, less enzyme enters the lysosome and can interact with saposin B.

**Figure 5:**
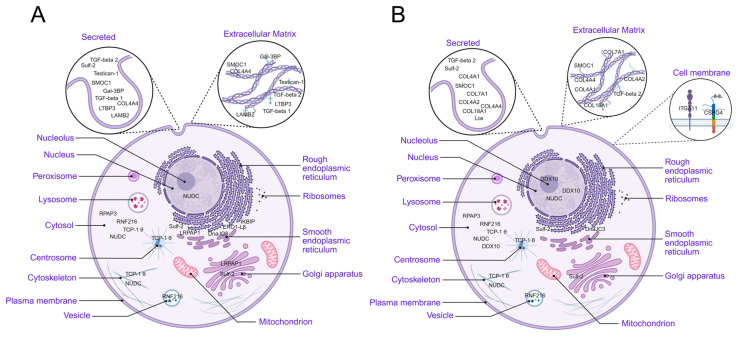
**Cellular localization of the top hit proteins for the AGAL-WT-(A) and AGAL-p.N215S-TurboID samples (B).** (**A**) Localization of the top AGAL-WT-TurboID hits, which are predominantly secreted or located at the ECM, with other proteins localized in the ER, Golgi, or cytosol. (**B**) Localization of the top AGAL-p.N215S-TurboID hits, also predominantly secreted or associated with the ECM, while other proteins are localized in the cytosol, nucleus, or cytoskeleton. Nine proteins overlap between the AGAL-WT- and AGAL-p.N215S-TurboID conditions. Detailed information on the individual proteins can be found in the supplemental material.

A significant proportion of the top hits identified for both AGAL-WT and AGAL-p.N215S are either components of the ECM or secreted proteins (Figure 5). These include collagens, integrins, and ECM remodeling proteins. Previous studies have already demonstrated the potential role of the ECM in the pathogenesis of FD [6]. A meta-analysis of published omics studies comparing urine and blood samples from treatment-naïve patients and ERT-treated patients with FD identified acute inflammatory responses, ECM remodeling, platelet function, and oxidative stress as altered central processes in treatment-naïve patients [6]. ECM remodeling was mainly attributable to collagen, fibronectin, and other proteins, such as LTBP2 and APP, and was partially regulated by microRNAs [6].

Markers of ECM turnover are also altered in FD. For example, one study including 29 patients (missense and nonsense variants) showed elevated MMP-9 serum levels, while TIMP-1 and TIMP-2 levels did not differ significantly [25]. These changes were associated with cardiac progression. Another study described elevated serum angiostatin levels in addition to MMP-9, which also indicates ECM turnover [26]. In this respect, a more recent study demonstrated that AGAL-deficiency was associated with a reduced endothelial glycocalyx [27]. Our identification of several ECM components as potential proximal AGAL proteins could support the hypothesis that ECM alterations in FD may be attributable, at least in part, to direct or indirect interactions between AGAL and ECM-associated proteins. This could also explain the risk of FD-related endothelial dysfunction. However, it remains unclear whether the identified ECM proteins represent true intracellular interaction partners of AGAL or merely reflect secretory pathway proximity labeling. This limitation needs to be addressed in further studies.

In addition to ECM proteins, proteins associated with ER stress and chaperones have also been identified. DNAJC3 is an ER-specific co-chaperone in complex with BiP. Verma et al., 2026, demonstrated an upregulation of HSP70 and associated adapter proteins in *GLA* KO mice and EA.hy926 *GLA* KO endothelial cells, indicating the involvement of ER stress and UPR signaling pathways [28]. However, no Hsp70 expression was detected in podocytes [28].

The interaction between AGAL and Gal-3BP has been demonstrated by co-IP. Gal-3BP is a multifunctional, highly glycosylated protein involved in cell adhesion and immune defense [29,30]. Furthermore, increased expression has been found in cancer, e.g., breast cancer [31]. The protein interacts with fibronectin, nidogen, and collagens IV, V, VI, which were also partially detected in our experiment [29]. Another study demonstrated the interaction between Gal-3BP and Sulf-2, which is one of our identified top hits [32]. Interestingly, a comparison between treatment-naïve and ERT-treated patients with FD showed an increased scavenger receptor activity in ERT-treated patients, which was attributable to the proteins Gal-3BP and tubulointerstitial nephritis antigen-like 1 [6]. Further experiments are now warranted to examine the role of AGAL in this context in more detail.

### 3.2. Specific Findings in the Late-Onset Cardiac p.N215S Variant

p.N215S is a disease-causing AGAL variant with severe clinical manifestations found primarily in the heart. Cardiac involvement may become as severe as in classic patients with FD, especially in males [33].

In the ER, three carbohydrate chains are attached to AGAL. The amino acid substitution at position p.N215 leads to a loss in glycosylation at this position [23]. Consequently, proper phosphorylation to mannose 6-phosphate cannot occur in the Golgi apparatus. This results in increased aggregation, retention in the ER, and finally protein degradation [23]. Due to its impact on folding and trafficking, this variant may have different pathways and interacting proteins compared with the WT AGAL.

In this study, a total of 411 proteins were identified as significantly enriched compared with the control. Of these, 152 proteins were significant in both groups (AGAL-WT and AGAL-p.N215S). AGAL-WT and AGAL-p.N215S were compared based on a combined score of fold change and adjusted *p*-value (Supplementary Figure S2B). Six proteins were significantly enriched compared with the control group and deviated from the diagonal line in Supplementary Figure S2B, which means that they were enriched to varying degrees. Such proteins are potentially relevant candidates for a direct functional comparison between AGAL-WT and AGAL-p.N215S.

The probable ATP-dependent RNA helicase DDX10 was more abundant in the AGAL-p.N215S-TurboID samples than in the AGAL-WT-TurboID samples. DDX10 is predominantly localized in the nucleolus, where it is involved in ribosome biogenesis [34]. After synthesis, AGAL translocates via the ER and the Golgi apparatus to the lysosomes. A direct physical interaction between these two proteins appears unlikely.

If the observed enrichment of DDX10 is not a side effect caused by overexpression, there still might be an indirect functional relationship. The p.N215S variant leads to misfolding and aggregation, resulting in increased retention and degradation in the ER [23]. This could trigger cellular stress responses affecting both ribosome biogenesis and the expression and stability of nucleolus-associated proteins, including DDX10. Increased levels of DDX10 in the AGAL-p.N215S-TurboID samples could reflect an indirect cellular adaptation rather than a specific interaction with AGAL. Further experiments are required to prove this hypothetical context.

### 3.3. Limitations

Proximity labeling with TurboID to identify new interaction partners has some limitations. These limitations primarily concern the identification of false-positive hits. TurboID has a short labeling time, which could increase specificity [35]. Nevertheless, this process can identify proteins that co-localize but do not directly interact. Potential endogenous biotin may biotinylate proteins prior to the external addition of biotin, thereby increasing labeling time. By using a biotin-poor medium, five biological replicates, and a control cell line (AGAL-SP-TurboID) for nonspecific interactions, background effects were kept to a minimum. The use of an immortalized cell line, such as AB8/13, might be a limitation. Labeling was performed in an artificial overexpression model. To minimize this risk of nonspecific biotinylation by protein overexpression, we used an inducible expression system, which allowed us to control the amount of expressed protein. In the current work, one candidate (Gal-3BP) was experimentally validated, while 410 proteins remain merely putative proximal proteins, which is a limitation. However, a full validation of all identified potentially interacting partners in the current work was not possible. Thus, the confirmation of potential interaction partners needs to be verified through additional experiments by our group and others who might explore potential candidates that could be relevant to their specific area of research in FD. The introduction of additional controls alongside the AGAL-SP-TurboID control, such as a specific control localized to the lysosome, would have been helpful in distinguishing between compartment-specific background signals. Additionally, fusion of the TurboID may influence AGAL, although an effect on enzyme activity has been ruled out. However, it may affect binding. Thus, the interaction of AGAL with potential interaction partners needs to be verified in additional experiments.

## 4. Methods and Materials

### 4.1. Cloning and Site-Directed Mutagenesis

For overexpression, the *GLA* coding sequence was cloned out of the vector pEGFP_*GLA* wild-type (WT) using restriction enzymes (EcoRI and BamHI) in frame into the pENTR_apol1-vB3 vector, already containing the TurboID gene (a gift from Thomas Weide, Institute for Molecular Nephrology, University Hospital Muenster). Ligation was performed using the T4 DNA ligase (EL0011, Thermo Fisher Scientific, Waltham, MA, USA), according to the manufacturer’s instructions, with incubation overnight at 4 °C. The resulting fusion gene was inserted into the destination vector pInducer21-puro (a gift from Thomas Weide, Institute for Molecular Nephrology, University Hospital Muenster) by Gateway cloning using LR Clonase^TM^ II enzyme mix (Thermo Fisher Scientific). Briefly, 50–150 ng of pENTR vector was mixed with 150 ng of the destination vector and 2 µL LR Clonase^TM^ II enzyme mix in TE buffer. The mixture was then incubated for 1 h at 25 °C. The reaction was terminated by adding Proteinase K solution and subsequent incubation of the sample for 10 min at 37 °C.

Site-directed mutagenesis was performed to generate the c.644A > G mutation into the *GLA*-WT gene using specific primers (forward: 5′-GTGGCCCTTTCAAAAGCCCAGTTATACAGAAATCCGACAGTAC-3′ and reverse: 5′-GTACTGTCGGATTTCTGTATAACTGGGCTTTTGAAAGGGCCAC-3′). After amplification, the template DNA was digested with DpnI. The consistency of constructs was controlled by sequencing.

### 4.2. Cell Culture

HEK293T cells were cultivated in Dulbecco’s modified Eagle medium (DMEM), supplemented with 10% FCS and 1% Penicillin/Streptomycin/Glutamine at 37 °C and 5% CO_2_. AB8/13 cells were cultured in Roswell Park Memorial Institute (RPMI) 1640 Medium, supplemented with 10% FCS and 1% Penicillin/Streptomycin/Glutamine/further supplements (including insulin–transferrin–sodium, sodium pyruvate, and MEM non-essential amino acid solution) at 30 °C and 5% CO_2_. The temperature was shifted to 37 °C when cells were seeded for experiments. To reduce background signals and nonspecific labelling, RPMI medium was replaced with biotin-free DMEM.

### 4.3. Generation of Stable Cell Lines

A lentiviral approach was chosen to introduce the pInducer21-puro vectors into AB8/13 cells, based on Schulze et al. 2014 [36]. In brief, HEK293T cells were transiently transfected with the pInducer21-puro construct and viral envelope-expressing plasmid pMD2.VSVG (Addgene #12259, a gift from Thomas Weide, Institute for Molecular Nephrology, University Hospital Muenster) and the viral packaging plasmid psPAx2 (Addgene #12260, a gift from Thomas Weide, Institute for Molecular Nephrology, University Hospital Muenster). Virus particle-containing supernatant was added to AB8/13 target cells after being filtered (0.45 µm), along with fresh medium containing polybrene (final concentration 8 µg/mL), in a 1:1 ratio. The medium was replaced with fresh virus particle-free medium, and the cells were regenerated for 24 h. The transduction was repeated once. The transduced cells were cultured as described above for the AB8/13 WT cells.

### 4.4. Induction of TurboID-Fusion Protein Expression and Biotin Labelling

TurboID-fusion protein expression was induced by adding doxycycline (125 ng/mL) for 24 h. For labeling, a 0.2 M biotin stock solution in DMSO was diluted to a final concentration of 50 µM in cell culture medium. The cells were incubated with biotin for 20 min at 37 °C. To stop labeling, the cells were washed three times with ice-cold PBS. Subsequently, the cells were lysed using RIPA buffer and frozen at −80 °C. Lysates were thawed and centrifuged to remove debris.

### 4.5. Streptavidin Pulldown

A total of 20 µL lysate was incubated with streptavidin-coated Dynabeads (11206D, Thermo Fisher Scientific) in PBS overnight at 4 °C, with rotation. Afterwards, beads were washed as previously described in Supplementary Table S1 [37]. All wash fractions were collected. After the final washing step, the beads were resuspended in an elution buffer and boiled at 95 °C for 5 min [37]. The supernatant was collected, and the remaining beads were subjected to a second elution in fresh elution buffer, as described above. All fraction samples were used for Western blot analysis.

### 4.6. Western Blot Analysis

The proteins were separated by SDS-PAGE and transferred onto a PVDF membrane for immunodetection. To minimize nonspecific antibody binding, the PVDF membrane was blocked with 5% milk powder/TBS-Tween (1%), or with 5% BSA/TBS-Tween when probed with streptavidin-HRP antibody. The membranes were incubated with primary antibodies diluted in 5% milk powder/TBS-Tween overnight at 4 °C, including anti-AGAL (ab168341, Abcam, Cambridge, UK), anti-BirA (TurboID) (AGR-AS20-4440, Agrisera, Vännäs, Sweden), and anti-vinculin (V9131, Sigma-Aldrich (Merck, Darmstadt, Germany) at 1:5.000. Unbound antibodies were removed by washing four times for 5 min each with TBS-Tween (1%). The membranes were subsequently incubated with a secondary antibody (Goat Anti-Rabbit IgG Antibody, HRP-conjugate (12-348, Merck Millipore, Billerica, MA, USA, 1:10.000) for 1 h at room temperature (RT). Streptavidin-HRP (434323, Thermo Fisher Scientific) diluted in 5% BSA/TBS-Tween was used to detect biotinylated proteins. The membrane was thoroughly washed with TBS-Tween (four times for 5 min each). For detection, the membranes were incubated with Clarity^TM^ Western ECL substrate (Bio-Rad, Hercules, CA, USA) for 5 min.

### 4.7. Immunofluorescence Staining

To control fusion protein expression and biotin labeling, immunofluorescence staining was performed. The cells were grown on a gelatin-coated glass coverslip to a confluence of 60–70%. The cells were treated with doxycycline and biotin, as described above. Doxycycline and/or biotin were omitted for the control cells. The cells were fixed with 4% paraformaldehyde/PBS for 10 min at RT and permeabilized with 0.2% Triton/PBS for 8 min. Afterwards, the cells were incubated in a blocking solution (5% BSA/PBS). Primary antibody staining was performed overnight at 4 °C (anti-AGAL ab168341 (Abcam) with anti-BirA (mutated/TurboID) AGR-AS20-4440 (Agrisera), 1:100). After washing, secondary antibody staining was performed (anti-Rabbit IgG AF488 18772-1ML-F, Sigma-Aldrich, NeutrAvidin AF647, 1:1000). Nuclei were stained with Hoechst33342 (H1399, Thermo Fisher Scientific) for 20 min. Finally, the cells were washed thoroughly and mounted in mowiol on object slides. The cells were imaged using fluorescence microscopy (Zeiss Axio Observer Z1, Oberkochen, Germany).

### 4.8. α-Galactosidase A Activity Measurement

AGAL enzyme activity [38] was measured using 4-methylumbelliferyl α-D-galactopyranoside (4-MUG) as the substrate. In brief, 5 µL of cell lysate was incubated with 4-MUG and the inhibitor of endogenous α-galactosidase B, N-acetylgalactosamine, under acidic conditions for 1 h at 37 °C [39]. The reaction was stopped using 0.5 M sodium carbonate. Fluorescence activity was measured at 460 nm. For absolute quantification of AGAL activity, a standard curve of defined concentrations of agalsidase beta (Sanofi, Gentilly, France) was included.

### 4.9. Preparation of Large-Scale Samples for Mass Spectrometric Analysis

To prepare the samples for mass spectrometric analysis, cells for each sample were cultured in three 10 cm culture dishes in DMEM for 24 h. Expression of the TurboID-fusion proteins was induced by adding doxycycline (125 ng/mL) for a further 24 h. Labeling was initiated by adding biotin (50 µM) for 20 min at RT, and terminated by washing the dishes three times with ice-cold PBS. Doxycycline and/or biotin were omitted for control samples, too. The cells were lysed in RIPA buffer supplemented with PhosSTOP (Roche, Basel, Switzerland), scraped, and the lysates were frozen at −80 °C. After thawing, the lysates were centrifuged to remove cell debris. For each cell line, 5 × 1 mg of total protein was incubated with streptavidin-coated Dynabeads in PBS overnight at 4 °C, with rotation. The beads were washed as described before. All wash fractions were collected. After the final wash step, the beads were resuspended in 500 µL PBS, 30 µL was taken for a control blot, and the remaining sample was stored at −80 °C.

### 4.10. Mass Spectrometric Analysis

Proteins were eluted from the beads by boiling them in an SDTB buffer (100 mM Tris pH 7.6, 4% SDS (*w*/*v*), 100 mM DTT + 2 mM biotin) for 5 min. Cold supernatant was prepared for MS analysis following the SP3 protocol, using TCEP and CAA for reduction and alkylation [40].

LC-MS/MS analysis was performed by using an EASY-nLC 1200 coupled to an Exploris 480 mass spectrometer (Thermo Fisher Scientific). Separation of peptides was performed on 20 cm frit-less silica emitters (CoAnn Technologies, Richland, WA, USA, 0.75 µm inner diameter), packed in-house with reversed-phase ReproSil-Pur C_18_ AQ 1.9 µm resin (Dr. Maisch, Ammerbuch-Entringen, Germany). The column was constantly kept at 50 °C. Peptides were eluted in 75 min by applying a segmented linear gradient of 0% to 98% solvent B (solvent A 0% ACN, 0.1% FA; solvent B 80% ACN, 0.1% FA) at a flow rate of 300 nL/min.

Mass spectra were acquired in data-dependent acquisition mode.

MS^1^ scans were acquired at an Orbitrap Resolution of 120,000 with a Scan Range (*m*/*z*) of 280–1500, a maximum injection time of 100 ms, and a normalized AGC Target of 300%. For fragmentation, only precursors with charge states 2–6 were considered. Up to 20 dependent scans were taken. For dynamic exclusion, the exclusion duration was set to 40, with a mass tolerance of +/− 10 ppm. The isolation window was set to 1.6 *m*/*z* with no offset. A normalized collision energy of 30 was used. MS^2^ scans were taken at an Orbitrap Resolution of 15,000, with a fixed first mass (*m*/*z*) = 100. The maximum injection time was 150 ms, and the normalized AGC target was 5%.

### 4.11. Data Analysis of Mass Spectrometric Analysis

The data were processed using Fragpipe 23.0 with MSFragger 4.3, IonQuant 1.11.11, and Philosopher 5.1.1, and searched against the Uniprot Reference Proteome for *homo sapiens*, including the TurboID constructs, common contaminants, and decoys. LFQ and MBR were enabled. All other settings were kept at default [41,42,43,44]. The reports were imported and further processed with R 4.0.0, quantifying protein groups by using the MaxLFQ intensities [45].

Missing values were imputed based on quantile regression per run (imputeLCMD version 2.1), and limma (version 3.46.0) was used for differential expression analysis by empirical Bayes moderation [46,47,48].

### 4.12. Further Data Analysis of Mass Spectrometric Analysis

For further data analysis, the following selection criteria were applied: contaminating proteins (e.g., albumin, casein) were excluded, proteins had to be enriched in at least 3 of the 5 samples examined under one condition, and an adj. *p*-value of <0.05, together with a log_2_ fold change of >1, were chosen as the threshold for significance [49,50]. For volcano plot visualization, only contamination proteins were excluded, and non-significant hits were still displayed. Python 3.13.7 was used for visualization. Functional enrichment analysis, including GO-term enrichment and pathway analysis, was carried out using the web tool g:profiler with predefined settings (Available online: https://biit.cs.ut.ee/gprofiler/gost (accessed on 11 March 2026)).

### 4.13. Co-Immunoprecipitations

AB8/13 were transiently transfected with a vector expressing AGAL-myc tag. The cells were seeded in 6-well plates and lysed using a co-IP lysis buffer (1% Triton, 0.5% NP-40, 150 mM NaCl, and 10 mM Tris-HCl (pH 7.4), 1 mM EDTA, a protease inhibitor, and a phosphatase inhibitor. Dynabeads Protein G (10003D, Thermo Fisher Scientific) were used for the co-IP, according to the protocol. In brief, the beads were incubated with an anti-myc tag antibody (#2276, Cell Signaling Technology, Inc., Danvas, MA, USA) for 30 min at RT. After washing, the cell lysate diluted in PBS was added, and the mixture was incubated for 2 h at 4 °C, with rotation. Subsequent elution was done by adding lysis buffer and Laemmli buffer, and heating to 95 °C.

## 5. Conclusions

In summary, TurboID has identified potential additional AGAL interaction partners that may be significant for the pathogenesis of FD. The identification of several ECM-associated proteins might strengthen the link between AGAL deficiency and impaired ECM turnover. Further experiments are needed to confirm interactions with other proteins and to determine involvement of AGAL in the identified processes.

## Data Availability

The original contributions presented in this study are included in the article/Supplementary Materials. Further inquiries can be directed to the corresponding authors.

## Supplementary Materials

The following supporting information can be downloaded at: https://www.mdpi.com/article/10.3390/ijms27125437/s1. References [51,52,53,54,55,56,57,58,59,60,61,62,63,64,65,66,67,68,69,70,71,72,73,74,75,76,77,78,79,80,81,82,83,84,85,86,87,88,89,90,91,92,93,94,95,96,97,98,99,100,101,102,103,104,105,106,107,108,109,110,111] are cited in the Supplementary Materials.
