# Supplementary material for: Identification of Potential Proteins Interacting with α-Galactosidase A to Analyze the Pathogenesis of Fabry Disease
Source: Int J Mol Sci. 2026 Jun 16;27(12):5437. doi: 10.3390/ijms27125437 (PMC13299219; doi:10.3390/ijms27125437)
Supplement: Supplementary file 1 [file ijms-27-05437-s001.zip › ijms-4363679-supplementary.pdf]

Supplementary Figures and Tables

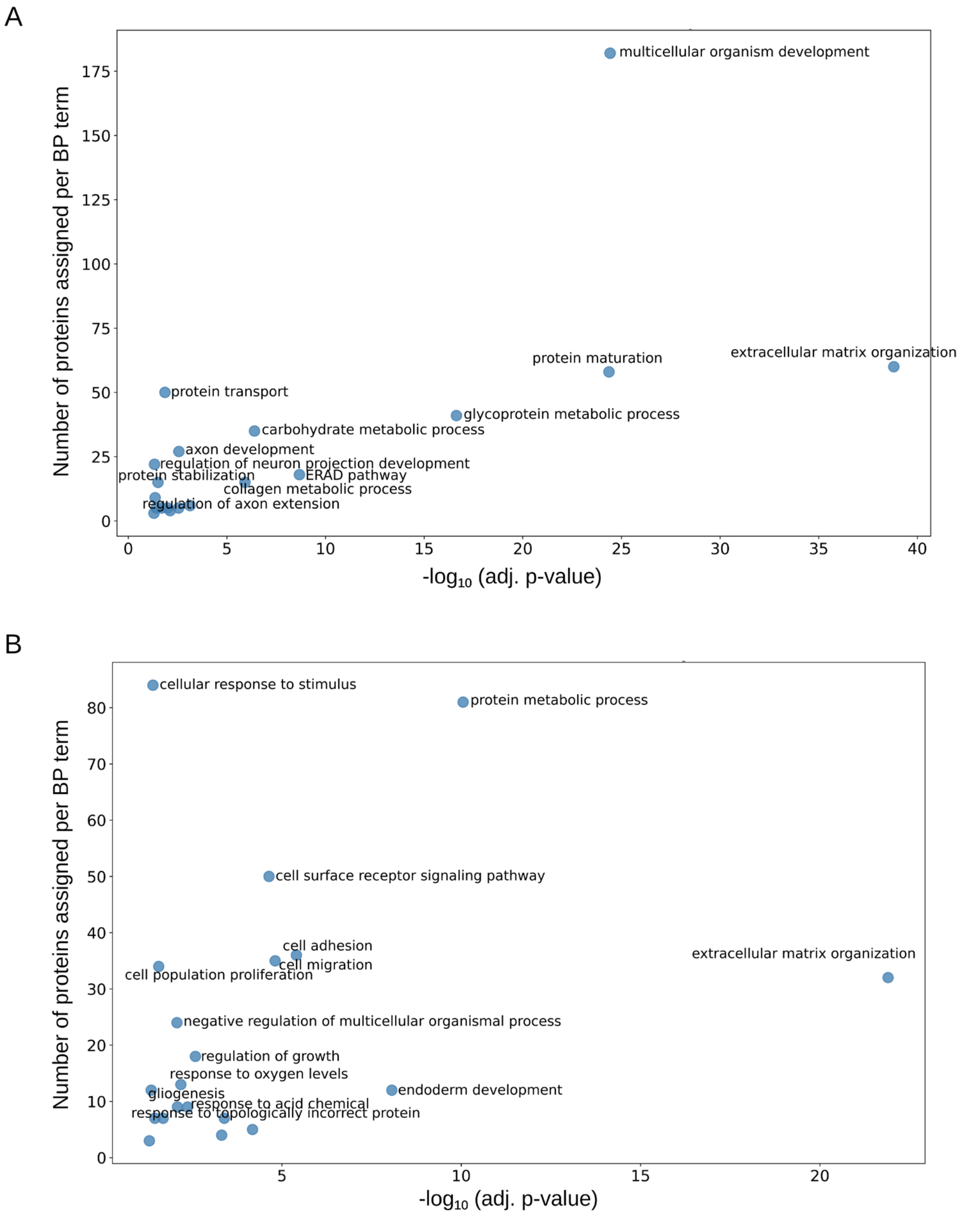

Supplementary Figure S1: Different protein enrichment and GO biological process analysis in AGAL-WT-TurboID and AGAL-p.N215S-TurboID samples. A) Number of proteins assigned per BP term of the AGAL-WT-TurboID samples in relation to -$\log_{10}$(adj. p-value). **B)** Number of proteins assigned per BP term of the AGAL-p.N215S-TurboID samples in relation to -$\log_{10}$(adj. p-value).

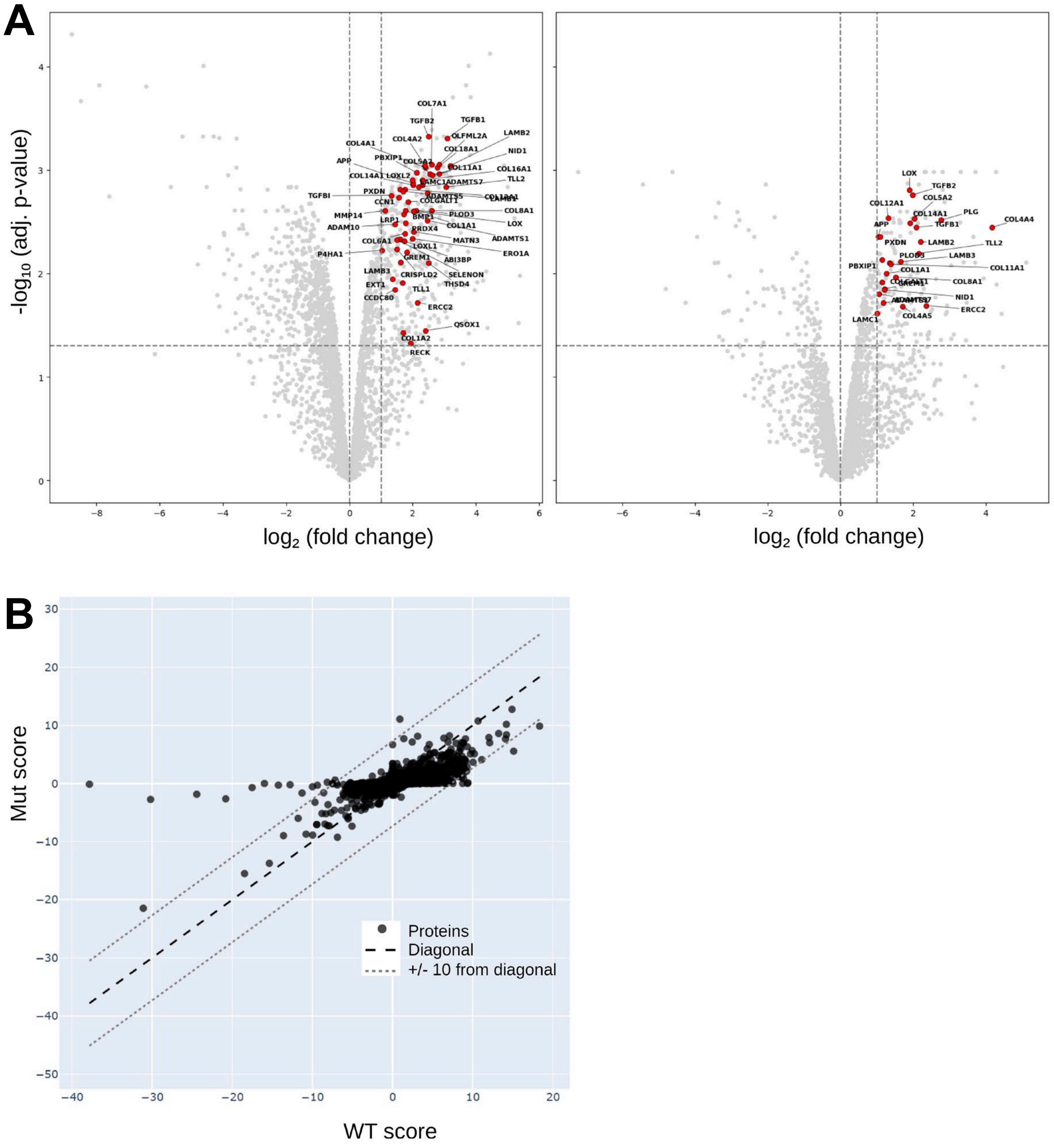

Supplementary Figure S2: Different protein enrichment and GO biological process analysis in AGAL-WT-TurboID and AGAL-p.N215S-TurboID samples. A) Volcano plots of identified proteins in AGAL-WT- (left) and AGAL-p.N215S-TurboID samples (right) compared to the AGAL-SP-TurboID control. Proteins assigned to the BP GO term *ECM organization* are highlighted. **B)** Comparison of proteins enriched in AGAL-WT- and AGAL-p.N215S-TurboID samples using a combined score for effect strength (fold change) and statistical significance (adjusted p-value, $\log_2(\text{fold change})\cdot(-\log_{10}(\text{adj. p-value}))$). High positive scores indicate a significantly stronger enrichment in the samples compared to the AGAL-SP-TurboID control; strong negative values indicate a significantly reduced enrichment. A score close to 0 means minor differences and no significance. Deviations from the diagonal (threshold ±10) indicate proteins that are selectively enriched in one of the two conditions (AGAL-WT- or AGAL-p.N215S-TurboID).

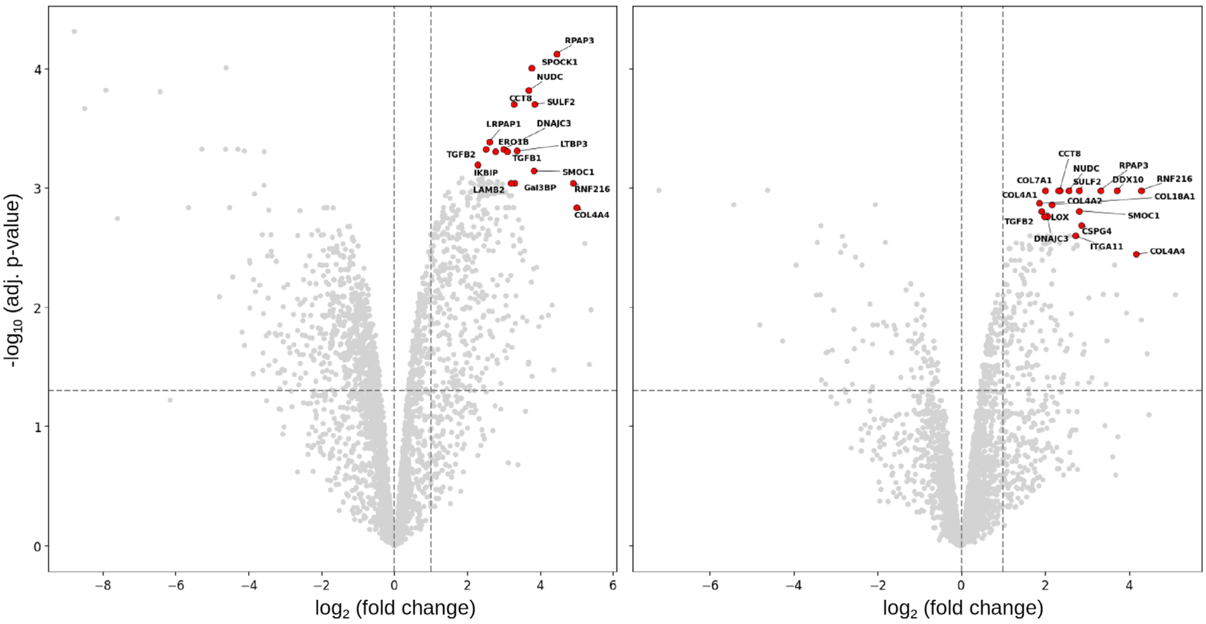

Supplementary Figure S3: Top hits of enriched proteins in AGAL-WT- and AGAL-p.N215S-TurboID samples. Volcano plots of identified proteins in AGAL-WT- (left) and AGAL-p.N215S-TurboID samples (right) compared to the AGAL-SP-TurboID control. Top hits proteins are highlighted.

**Supplementary Table S1: Washing protocol for streptavidin bead-bound proteins.**		
**Component**	**Volume**	**Incubation duration [RT]**
PBS buffer	1 ml	2 min
PBS buffer	1 ml	2 min
1 M KCl	1 ml	2 min
0,1 M Na_2CO_3	1 ml	10 s
2 M Urea in 10 mM Tris-HCl (pH 8.0)	1 ml	10 s
PBS buffer	1 ml	2 min
PBS buffer	1 ml	2 min

Supplementary Table S2: Top hits of enriched proteins in AGAL-WT-TurboID samples.

Protein	Gene	Overview	Subcellular location	Interacting proteins	Size ca. (kDa)	References	Adj. p-value	Log_2 fold change
**Nuclear migration protein nudC**	NUDC	Necessary for cytokinesis and cell proliferation, Colocalize with tubulin and dynein and with microtubule organizing center, ubiquitous expression, essential for actin organization and dynamics by stabilizing cofilin1	Cytoplasm, cytoskeleton, spindle, nucleus	PAFAH1B1, PLK1, DCDC1, EML4, FBXW2, WRAP73, WDFY2, DYNC2I1, POC1B, POC1A, ATN1, CRYAA, DNAJA2, GIPC1, EML1	38	(1, 2)<br>uniport (Q9Y266)	0.0002	3.69
**DnaJ homolog subfamily C member 3**	DNAJC3	Synonyms: P58IPK and PRKRI, participates in the unfolded protein response during ER stress, inhibits phosphorylation of eukaryotic initiation factor 2α (eIF2α), downregulation of the PERK/eIF2α/ATF4 pathway, upregulated by ATF6 during ER stress, downregulated by DNAJB1, trimeric complex with DNAJB1 and HSPA8 (co-chaperone of HSPA8/HSC70)	ER	EIF2AK2, DNAJB1, HSPA8	58	(3–5)<br>uniport (Q13217)	0.0005	2.98
**ERO1-like protein beta**	ERO1B	Sulfhydryl Oxidase, part of disulfide bond formation in ER (reoxidizes the oxidase PDI, which in turn oxidizes disulfide bridges in substrate proteins), transcription induced by UPR	ER membrane	P4HB, ERP44	54	(6, 7)<br>uniport (Q86YB8)	0.0005	2.76
**Alpha-2-macroglobulin receptor-associated protein**	LRPAP1	Molecular chaperone for ldl receptor-related proteins (e.g. LRP1), inhibits multiple ligands from binding to ldl-receptors, disease: myopia 23 (ocular refractive error), post-translational modification: N-glycosylated	ER, ER-Golgi intermediate compartment lumen, golgi, golgi lumen, Endosome lumen, cell surface	LRP1/alpha-2-macroglobulin receptor heavy and light chains, LRP2/Megalin, LRP1B, LDLR, SORL1, SORT1, LRP8, ASPH, VLDLR, APP	39	(8–10)<br>uniport (P30533)	0.0004	2.61
**Extracellular sulfatase Sulf-2**	SULF2	Arylsulfatase activity (aryl sulfate + H2O = a phenol + sulfate + H^+, optimum pH 7.0-8.0), hydrolysis of specific groups of heparan sulfate and keratan sulfate (leads to changed binding of growth factors and promote or inhibit signalling)	ER, Golgi, Cell surface and secreted form	BioGRID: 126 interactors	100	(11)<br>uniport (Q8IWU5)	0.0002	3.84
**T-complex protein 1 subunit theta**	CCT8	Component of the chaperonin-containing T-complex (TRiC) (folding of actin, tubulin and other proteins), may play a role in the assembly of BBSome (involved in ciliogenesis), overexpressed in many cancers	Cytoplasm, cytoskeleton, microtubule organizing center, centrosome, cilium basal body	SNX12, MAGEB6, CDHR3, BRME1, TUBA1B, BAG6, HTT, CCT6A, TCPA, LASP1	59	(12)<br>uniport (P50990)	0.0002	3.27
**E3 ubiquitin-protein ligase RNF216**	RNF216	Protein ubiquitination, accepts ubiquitin from specific E2 ubiquitin-conjugating enzymes, and then transfers it to substrates promoting their ubiquitination, regulates Toll-like receptors (immune system), down-regulates NF-kappa-B and blocked IRF3 activation as well as beta interferon production, plays a role in regulation of autophagy by ubiquitination, highest level of expression in testis and peripheral blood leukocytes	Cytoplasm, cytoplasmic vesicle, clathrin-coated vesicle	UBE2L3, UBE2L6, TRAF3, TLR3, TLR4, TLR5, TLR9, UBAC1, OPTN, FAM168A, UBASH3B, ZFAND2A, MTURN, OTU1, MORC3, UBASH3A, UBP5, TNFAIP3, CAPN15, KHNYN	99	(13, 14)<br>uniport (Q9NWF9)	0.0009	4.90

Supplementary Table S3 (continued): Top hits of enriched proteins in AGAL-WT-TurboID samples.

**RNA polymerase II-associated protein 3**	RPAP3	Component of the R2TP complex (co-chaperone of HSP90; participates in gene expression, translation, co-translational complex assembly, and posttranslational protein complex formation), connecting the RNA polymerase II enzyme and chaperone/scaffolding protein, recruiting client proteins, component of the PAQosome complex (as a part of R2TP complex)	Cilium, cytosol	PIH1D1, TSC1, TSC2, PRPF8, EFTUD2, POLR3A, PFDN2, UXT, DNAAF10, TERF1, HSP90AB1, HSP90AA1, URI1	76	(15, 16) uniport (Q9H6T3)	0.0001	4.45
**Collagen alpha-4(IV) chain**	COL4A4	As a key structural component of the glomerular basement membrane essential for stability, integrity and functionality, part of COL4A3/4/5 trimer, forms a network with laminins, proteoglycans and nidogen, associated disease: Alport syndrome 2 (symptoms: progressive glomerulonephritis, persistent haematuria, hearing loss, ocular defects, tubulointerstitial atrophy), contain various cysteine residues (important for inter- and intramolecular disulfide bond formation), associated with LAMB2	Secreted, extracellular space, extracellular matrix, basement membrane	COL4A3, COL4A5	164	(17, 18)	0.0015	5.01
**Inhibitor of nuclear factor kappa-B kinase-interacting protein**	IKBIP	Target of p53/TP53, pro-apoptotic function, same promotor as APAF1, regulates negatively the activation of NF-κB	ER membrane	WASHC3, TFIP11, NOD2, BBLN, SMARCD1, TPM3, NUP62, KRT15, MYOG, LCA5L, SNAPIN, CDK4	39	(19, 20)	0.0006	2.27
**Laminin subunit beta-2**	LAMB2	Part of the glycoprotein laminin (three polypeptide chains alpha, beta, gamma, cross-shaped structure), laminin mediates cell adhesion, differentiation and migration, part of basement membranes, highly expressed in glomerular basement membrane, mutations in LAMB2 can lead to Pierson syndrome (congenital nephrotic syndrome with retinal and neuromuscular defects), part of podocyte-specific ECM protein isoforms (together with agrin and collagen type IV-4 (COL4A4)	Extracellular matrix, extracellular space, secreted	Laminin as a complex interact with Integrin (found in our experiment: ITGB5, ITGA3, ITGA11, ITGAV, ITGB3, ITGB1, ITGB4, ITGA5, ITGA2, ITGA6) and dystroglycan	196	(21, 18, 22) uniport (P55268)	0.0009	3.19
**Galectin-3-binding protein**	LGALS3 BP	Glycoprotein, involved in β1 integrins mediated cell adhesion, Synonym M2BP, binds to galectins 1, 3, and 7, fibronectin, and collagen IV, V, and VI (COL4A1, COL5A1, COL6A1), connecting a complex consisting of SMYLE, AKAP9 and CKD5RAP2 to microtubule-nucleating complex (γ-TuRC)	Secreted, extracellular matrix, extracellular space	SAMHD1, FBXO6, BCL2L14, TWNK, VHL, MX1, CFTR, VAMP5, TNFRSF10A	65	(23–25) uniport (Q08380)	0.0009	3.30
**Latent-transforming growth factor beta-binding protein 3**	LTBP3	Key regulator of transforming growth factor beta (TGFB1, TGFB2 and TGFB3), controls inactivation of TGFβ in extracellular space, interacts with latency-associated peptide (LAP), belong to the LTBP/fibrillin-family (fibrillin-1 and -2 are also members), associated with SMAD pathway (activation of SMAD leads to their translocation to the nucleus and expression of genes)	Secreted, extracellular space, extracellular matrix	FBN1 BioGRID: 51 interactors (high throughput)	139	(26–28) uniport (Q9NS15)	0.0005	3.36

Supplementary Table S4 (continued): Top hits of enriched proteins in AGAL-WT-TurboID samples.

**Transforming growth factor beta-1 proprotein**	TGFB1	Precursor of LAP and TGFβ1 chains (form the active subunit of TGFβ1), cleavage of proprotein in Golgi, TGFβ1 remain inactive in ECM, activation of TGFβ1 by LTBP1, LRRC32 and LRRC33, interaction with integrins (ITGAV:ITGB6 or ITGAV:ITGB8) leads to release of active TGFβ1, after activation and release TGFβ1 binds to TGFBR1 and TGFBR2, which transduce signal, involved in SMAD pathway, can induce epithelial-to-mesenchymal transition and cell migration	Secreted, extracellular matrix, extracellular space	KRTAP4-11, LCE3D, CATSPER1, KRTAP1-3, ADAMTSL4, LCE2C, LCE3B, LCE3A, LCE1A, LCE1D, LCE1C, LTBP1, DIP2A, LRRC32, FSTL1, POU4F2, KRTAP1-1, TGFBR3, KRTAP10-8, MEOX2, HOXA1, TGFBR2, TGFBR1, TNXB, ENG, PSG1, THBS1, P4HB, APP, LPXN, LCE2B, CYSRT1, FCHO1	44	(29) uniport (P01137)	0.0005	3.09
**Transforming growth factor beta-2 proprotein**	TGFB2	Regulation of cell growth, differentiation and synthesis, degradation and remodelling of ECM, precursor of LAP and TGFβ2 chains, which form the active subunit of TGFβ2, cleavage of proprotein in golgi, LAP and TGFβ2 remain non-covalent-bound, TGFβ2 remain thus inactive in ECM, activation of TGFβ2 by LTBP1 and LRRC32, after activation: release of LAP, and TGFβ2 binds to TGFBR1 and TGFBR2 and transduce signal	LAP: secreted, extracellular space, extracellular matrix TGFβ2: secreted	ASPN, LRRC32/GARP, NREP	48	(26) uniprot (P61812)	0.0005	2.50
**SPARC-related modular calcium-binding protein 1**	SMOC1	Glycoprotein, conformation is calcium-dependend, important for eye and limb development, potential regulator of osteoblast differentiation, associated disease: Ophthalmoacromelic syndrome (ocular anomalies and limb anomalies), N-glycosylated, same protein family as SPOCK1, inhibit downstream targets of the TGFβ- family member BMP2 including pSMAD1/5	Secreted, extracellular space, extracellular matrix, basement membrane	NR4A3, NOTCH2NLA, KRTAP1-1, KRTAP10-8, TCF4, VGLL3, CYSRT1	48	(30–32) uniprot (Q9H4F8)	0.0007	3.82
**Testican-1**	SPOCK1	Highly conserved multidomain proteoglycan, cell-cell and cell-matrix interactions, may play a role in diverse neuronal processes within the central nervous system, cancer related, role in lipid metabolism, downregulation of SPOCK1 seemed to affect Wnt signalling, inhibition of membrane-type matrix metalloproteinase	Secreted, ECM, extracellular space	CTSL, TGF-β, BCL2L13, CREB3L1	49	(33, 34) uniport (Q08629)	0.0001	3.77

Blue: detected in our experiment (present in at least one of the probes (AGAL-WT-, AGAL-p.N215S-, and/or AGAL-SP-TurboID).
Green: significantly increased in the AGAL-WT-TurboID samples compared to AGAL-SP-TurboID samples.
Yellow: significantly increased in the AGAL-p.N215S-TurboID samples compared to the control probe.
Orange: significantly increased in the AGAL-WT- and AGAL-p.N215S-TurboID samples compared to the AGAL-SP-TurboID samples.

Supplementary Table S3: Top hits of enriched proteins in AGAL-p.N215S-TurboID samples.

Protein	Gene	Overview	Subcellular location	Interacting proteins (gene names)	Size ca. (kDa)	References	Adj. p-value	Log_2 fold change
**Nuclear migration protein nudC**	NUDC	Necessary for cytokinesis and cell proliferation, co-localize with tubulin and dynein and with microtubule organizing center, ubiquitous expression, essential for actin organization and dynamics by stabilizing cofilin1	Cytoplasm, cytoskeleton, spindle, nucleus	PAFAH1B1, PLK1, DCDC1, EML4, FBXW2, WRAP73, WDFY2, DYNC2I1, POC1B, POC1A, ATN1, CRYAA, DNAJA2, GIPC1, EML1	38	(1, 2)<br>uniprot (Q9Y266)	0.0010	2.57
**DnaJ homolog subfamily C member 3**	DNAJC3	Synonyms: P58IPK and PRKRI, participates in the unfolded protein response during ER stress, inhibits phosphorylation of eukaryotic initiation factor 2α (eIF2α), downregulation of the PERK/eIF2α/ATF4 pathway, upregulated by ATF6 during ER stress, downregulated by DNAJB1, trimeric complex with DNAJB1 and Hsc70 (co-chaperone of Hsc70)	ER	EIF2AK2, DNAJB1, HSPA8	58	(3–5)<br>uniprot (Q13217)	0.0017	2.06
**Probable ATP-dependent RNA helicase DDX10**	DDX10	Synonyms: DEAD box protein 10, helicase activity, promotes AIM2-induced inflammasome activation, increases the production of type I interferon, negatively affects infection of PRRSV, related to a.o. colorectal cancer cells, lung carcinoma and oral squamous cell carcinoma, might affect MAPK pathway	Cytoplasm, nucleus, nucleolus	AIM2, SCNA, SQSTM1, RPL35, Rab27b	101	(35–39)<br>uniport (Q13206)	0.0010	3.72
**Collagen alpha-1(IV) chain**	COL4A1	One of six type IV collagen isoforms, part of collagen IV network, forms meshwork with laminins, proteoglycans and nidogen, C-terminal NC1 domain (Arresten) is an angiogenesis inhibitor (inhibits endothelial cell migration, proliferation and tube formation), variants of this genes lead to several diseases, cysteine residues are important for inter- and intracellular disulfide bonding, hydroxylated prolines and lysines and glycosylated lysines, N-glycosylation	Secreted, extracellular matrix, extracellular space, basement membrane		161	(40–42)<br>uniprot (P02462)	0.0010	1.99
**Extracellular sulfatase Sulf-2**	SULF2	Arylsulfatase activity (aryl sulfate + H2O = a phenol + sulfate + H^+, optimum pH 7.0-8.0), hydrolysis of specific groups of heparan sulfate and keratan sulfate (leads to changed binding of growth factors and promote or inhibit signalling)	ER, Golgi, Cell surface and secreted form	BioGRID: 126 interactors	100	(11)<br>uniport (Q8IWU5)	0.0010	2.81
**T-complex protein 1 subunit theta**	CCT8	Component of the chaperonin-containing T-complex (TRiC) (folding of actin, tubulin and other proteins), may play a role in the assembly of BBSome (involved in ciliogenesis), overexpressed in many cancers	Cytoplasm, cytoskeleton, microtubule organizing center, centrosome, cilium basal body	SNX12, MAGEB6, CDHR3, BRME1, TUBA1B, BAG6, HTT, CCT6A, TCPA, LASP1	59	(12)<br>uniprot (P50990)	0.0010	2.35

Supplementary Table S3 (continued): Top hits of enriched proteins in AGAL-p.N215S-TurboID samples.

**E3 ubiquitin-protein ligase RNF216**	RNF216	Protein ubiquitination, accepts ubiquitin from specific E2 ubiquitin-conjugating enzymes, and then transfers it to substrates promoting their ubiquitination, regulates Toll-like receptors (immune system), down-regulates NF-kappa-B and blocked IRF3 activation as well as beta interferon production, plays a role in regulation of autophagy by ubiquitination, highest level of expression in testis and peripheral blood leukocytes	Cytoplasm, cytoplasmic vesicle, clathrin-coated vesicle	UBE2L3, UBE2L6, TRAF3, TLR3, TLR4, TLR5, TLR9, UBAC1, OPTN, FAM168A, UBASH3B, ZFAND2A, MTURN, OTU1, MORC3, UBASH3A, UBP5, TNFAIP3, CAPN15, KHNYN	99	(13, 14)<br>uniport (Q9NWF9)	0.0010	4.28
**RNA polymerase II-associated protein 3**	RPAP3	Component of the R2TP complex (co-chaperone of Hsp90; participates in gene expression, translation, co-translational complex assembly, and posttranslational protein complex formation), connecting the RNA polymerase II enzyme and chaperone/scaffolding protein, recruiting client proteins, component of the PAQosome complex (as a part of R2TP complex)	Cilium, cytosol	PIH1D1, TSC1, TSC2, PRPF8, EFTUD2, POLR3A, PFDN2, UXT, DNAAF10, TERF1, HSP90AB1, HSP90AA1, URI1	76	(15, 16)<br>uniprot (Q9H6T3)	0.0010	3.31
**Collagen alpha-4(IV) chain**	COL4A4	As a key structural component of the glomerular basement membrane essential for stability, integrity and functionality, part of COL4A3/4/5 trimer, forms a network with laminins, proteoglycans and nidogen, associated disease: Alport syndrome 2 (symptoms: progressive glomerulonephritis, persistent haematuria, hearing loss, ocular defects, tubulointerstitial atrophy), contain various cysteine residues (important for inter- and intramolecular disulfide bond formation), associated with LAMB2, N-glycosylated	Secreted, extracellular space, extracellular matrix, basement membrane	COL4A3, COL4A5	164	(17, 18)	0.0036	4.16
**Collagen alpha-1(VII) chain**	COL7A1	Part of epithelial basement membrane, interacts with type IV collagen, protease inhibitor, N- and O-glycosylation, Hydroxylation of prolines, inter- and intramolecular disulfide bonds, TANGO1 is important for export from the ER	Secreted, extracellular space, extracellular matrix, basement membrane	MIA3	295	(43)<br>uniport (Q02388)	0.0010	2.32
**Collagen alpha-2(IV) chain**	COL4A2	Part of glomerular basement membranes, meshwork with laminins, proteoglycans and nidogen, component of the α1α1α2 heterotrimer, C-terminal NC1 domain (canstatin) inhibits angiogenesis and tumor cell activity, interaction between canstatin and αvβ3- and αvβ5-integrin receptor, canstatin induced apoptosis signalling, N-glycosylated, inter- and intramolecular disulfide bonds,	Secreted, extracellular space, extracellular matrix, basement membrane	αvβ5 integrin, alphavbeta3 integrin, nidogen, fibronectin, Heparansulfat-Proteoglycan, heparin	168	(44–46)<br>uniport (P08572)	0.0014	1.85
**Collagen alpha-1(XVIII) chain**	COL18A1	Cleaved into endostatin and C-terminal non-collagenous (NC1) region by cathepsin L, multiple triple helix (motif: Gly-X-Y), Endostatin-, Rac-, Cdc42- and MAPK pathway-dependent involvement of NC1 region in endothelial and non-endothelial proliferation, migration and apoptosis, endostatin is an inhibitor of angiogenesis, N- and O-glycosylation	Secreted, extracellular space, extracellular matrix, basement membrane	VEGF-R2, αvβ1, αvβ5 and αvβ3 integrins, glypican3, fibulin1, fibulin2, perlecan, MMP-2, Tropomyosin, nidogen2	178	(47–52)	0.0014	2.16

Supplementary Table S3 (continued): Top hits of enriched proteins in AGAL-p.N215S-TurboID samples.								
**Protein-lysine 6-oxidase**	LOX	Oxidative modification (deamination) of lysine residues in precursors of collagen and elastin, cross-links collagen and elastin, leading to the formation of collagen fibrils, LOX precursor is cleaved by BMP1 and ADAMTS2/14, which alters its binding activity to collagen	Secreted, extracellular space	MFAP4	50	(53, 54) uniport (P28300)	0.0016	1.90
**Chondroitin sulfate proteoglycan 4**	CSPG4	Cell proliferation and migration, receptor for collagen alpha 2(VI), binds growth factors, involved in α4β1 integrin-mediated adhesion, may be involved in activation of Rho-family GTPase Cdc42, may bind type II collagen, type V collagen, tenascin and laminin, activation of MAPK/ERK pathway, O-glycosylated	Cell membrane, cell surface, apical cell membrane	COL5A1, COL6A2, ITGB1, ITGA3, tenascin, MUPP1, Plasminogen, Angiostatin, growth factors (bFGF, PDGF-AA)		(55–61) uniprot (Q6UVK1)	0.0021	2.87
**Transforming growth factor beta-2 proprotein**	TGFB2	Regulation of cell growth, differentiation and synthesis, degradation and remodeling of ECM, precursor of LAP and TGFβ2 chains, which form the active subunit of TGFβ2, cleavage of proprotein in Golgi, LAP and TGFβ2 remain non-covalent-bound, TGFβ2 remain thus inactive in ECM, activation of TGFβ2 by LTBP1 and LRRC32 protein, after activation: release of LAP, and TGFβ2 binds to TGFBR1- and TGFBR2-protein and transduce signal	LAP: secreted, extracellular space, extracellular matrix TGFβ2: secreted	ASPN, LRRC32/GARP, NREP	48	(26) uniprot (P61812)	0.0017	1.98
**SPARC-related modular calcium-binding protein 1**	SMOC1	Glycoprotein, conformation is calcium-dependend, important for eye and limb development, potential regulator of osteoblast differentiation, associated disease: Ophthalmoacromelic syndrome (ocular anomalies and limb anomalies), N-glycosylated, same protein family as Testican-1, inhibit downstream targets of the TGFβ- family member BMP2 including pSMAD1/5	Secreted, extracellular space, extracellular matrix, basement membrane	NR4A3, NOTCH2NLA, KRTAP1-1, KRTAP10-8, TCF4, VGLL3, CYSRT1	48	(30–32) uniprot (Q9H4F8)	0.0016	2.82
**Integrin alpha-11**	ITGA11	Part of heterodimer Integrin α11β1, receptor for collagen, important for collagen matrix and proliferation, FAK-dependent activation of MAPK/ERK pathway, internalized by multiple ways including nonclathrin- and caveolin-1 structures, associated with GTPases Rab21, N-glycosylated	Cell membrane	Rab21, collagen, PDGFRβ	133	(62–64) uniprot (Q9UKX5)	0.0025	2.72

Blue: detected in our experiment (present in at least one of the probes (AGAL-WT-, AGAL-p.N215S-, and/or AGAL-SP-TurboID).
Green: significantly increased in the AGAL-WT-TurboID samples compared to AGAL-SP-TurboID samples.
Yellow: significantly increased in the AGAL-p.N215S-TurboID samples compared to the control probe.
Orange: significantly increased in the AGAL-WT- and AGAL-p.N215S-TurboID samples compared to the AGAL-SP-TurboID samples.

Supplementary References

1. Gross AK, Sun J, Garner MA. The absence of NUDC in cone photoreceptors causes protein mislocalization and disrupts the scotopic electroretinographic b-wave. Invest Ophthalmol Vis Sci 2025; 66(8):6003.

2. Zhang C, Zhang W, Lu Y, Yan X, Yan X, Zhu X et al. NudC regulates actin dynamics and ciliogenesis by stabilizing cofilin 1. Cell Res 2016; 26(2):239–53.

3. Synofzik M, Ilg W. Motor training in degenerative spinocerebellar disease: ataxia-specific improvements by intensive physiotherapy and exergames. Biomed Res Int 2014; (1):583507.

4. van Huizen R, Martindale JL, Gorospe M, Holbrook NJ. P58IPK, a novel endoplasmic reticulum stress-inducible protein and potential negative regulator of eIF2alpha signaling. J Biol Chem 2003; 278(18):15558–64.

5. Yan W, Frank CL, Korth MJ, Sopher BL, Novoa I, Ron D et al. Control of PERK eIF2alpha kinase activity by the endoplasmic reticulum stress-induced molecular chaperone P58IPK. Proc Natl Acad Sci USA 2002; 99(25):15920–5.

6. Dias-Gunasekara S, Gubbens J, van Lith M, Dunne C, Williams JAG, Kataky R et al. Tissue-specific expression and dimerization of the endoplasmic reticulum oxidoreductase Ero1beta. J Biol Chem 2005; 280(38):33066–75.

7. Anelli T, Alessio M, Mezghrani A, Simmen T, Talamo F, Bachi A et al. ERp44, a novel endoplasmic reticulum folding assistant of the thioredoxin family. EMBO J 2002; 21(4):835–44.

8. Wagner MC, Sandoval RM, Yadav SPS, Campos SB, Rhodes GJ, Phillips CL et al. Lrpap1 (RAP) Inhibits Proximal Tubule Clathrin Mediated and Clathrin Independent Endocytosis, Ameliorating Renal Aminoglycoside Nephrotoxicity. Kidney360 2023; 4(5):591–605.

9. Bu G, Geuze HJ, Strous GJ, Schwartz AL. 39 kDa receptor-associated protein is an ER resident protein and molecular chaperone for LDL receptor-related protein. EMBO J 1995; 14(10):2269–80.

10. Aldahmesh MA, Khan AO, Alkuraya H, Adly N, Anazi S, Al-Saleh AA et al. Mutations in LRPAP1 are associated with severe myopia in humans. Am J Hum Genet 2013; 93(2):313–20.

11. Rosen SD, Lemjabbar-Alaoui H. Sulf-2: an extracellular modulator of cell signaling and a cancer target candidate. Expert Opin Ther Targets 2010; 14(9):935–49.

12. Liao Q, Ren Y, Yang Y, Zhu X, Zhi Y, Zhang Y et al. CCT8 recovers WTp53-suppressed cell cycle evolution and EMT to promote colorectal cancer progression. Oncogenesis 2021; 10(12):84.

13. Chuang T-H, Ulevitch RJ. Triad3A, an E3 ubiquitin-protein ligase regulating Toll-like receptors. Nat Immunol 2004; 5(5):495–502.

14. Nakhaei P, Mesplede T, Solis M, Sun Q, Zhao T, Yang L et al. The E3 ubiquitin ligase Triad3A negatively regulates the RIG-I/MAVS signaling pathway by targeting TRAF3 for degradation. PLoS Pathog 2009; 5(11):e1000650.

15. Martino F, Pal M, Muñoz-Hernández H, Rodríguez CF, Núñez-Ramírez R, Gil-Carton D et al. RPAP3 provides a flexible scaffold for coupling HSP90 to the human R2TP co-chaperone complex. Nat Commun 2018; 9(1):1501.

16. Lynham J, Houry WA. The Multiple Functions of the PAQosome: An R2TP- and URI1 Prefoldin-Based Chaperone Complex. Adv Exp Med Biol 2018; 1106:37–72.

17. Pöschl E, Schlötzer-Schrehardt U, Brachvogel B, Saito K, Ninomiya Y, Mayer U. Collagen IV is essential for basement membrane stability but dispensable for initiation of its assembly during early development. Development 2004; 131(7):1619–28.

18. Chan GC, Eng DG, Miner JH, Alpers CE, Hudkins K, Chang A et al. Differential expression of parietal epithelial cell and podocyte extracellular matrix proteins in focal segmental glomerulosclerosis and diabetic nephropathy. Am J Physiol Renal Physiol 2019; 317(6):F1680-94.

19. Hofer-Warbinek R, Schmid JA, Mayer H, Winsauer G, Orel L, Mueller B et al. A highly conserved proapoptotic gene, IKIP, located next to the APAF1 gene locus, is regulated by p53. Cell Death Differ 2004; 11(12):1317–25.

20. Wu H, Liu H, Zhao X, Zheng Y, Liu B, Zhang L et al. IKIP Negatively Regulates NF-κB Activation and Inflammation through Inhibition of IKKα/β Phosphorylation. J Immunol 2020; 204(2):418–27.

21. Funk SD, Lin M-H, Miner JH. Alport syndrome and Pierson syndrome: Diseases of the glomerular basement membrane. Matrix Biol 2018; 71:250–61.

22. Kikkawa Y, Hashimoto T, Takizawa K, Urae S, Masuda H, Matsunuma M et al. Laminin β2 variants associated with isolated nephropathy that impact matrix regulation. JCI insight 2021; 6(6):e145908.

23. White M. Linking Fibrosis and Cancer through the Differentiation of Fibrocytes [Doctoral dissertation].

24. Sasaki T, Brakebusch C, Engel J, Timpl R. Mac-2 binding protein is a cell-adhesive protein of the extracellular matrix which self-assembles into ring-like structures and binds beta1 integrins, collagens and fibronectin. EMBO J 1998; 17(6):1606–13.

25. Bouguenina H, Salaun D, Mangon A, Muller L, Baudelet E, Camoin L et al. EB1-binding-myomegalin protein complex promotes centrosomal microtubules functions. Proc Natl Acad Sci USA 2017; 114(50):E10687-96.

26. Penttinen C, Saharinen J, Weikkolainen K, Hyytiäinen M, Keski-Oja J. Secretion of human latent TGF-beta-binding protein-3 (LTBP-3) is dependent on co-expression of TGF-beta. J Cell Sci 2002; 115(17):3457–68.

27. Saharinen J, Keski-Oja J. Specific sequence motif of 8-Cys repeats of TGF-beta binding proteins, LTBPs, creates a hydrophobic interaction surface for binding of small latent TGF-beta. Mol Biol Cell 2000; 11(8):2691–704.

28. Attisano L, Wrana JL. Smads as transcriptional co-modulators. Curr Opin Cell Biol 2000; 12(2):235–43.

29. Zhao K, Zhang S, Song X, Yao Y, Zhou Y, You Q et al. Gambogic acid suppresses cancer invasion and migration by inhibiting TGFβ1-induced epithelial-to-mesenchymal transition. Oncotarget 2017; 8(16):27120–36.

30. Vannahme C, Gösling S, Paulsson M, Maurer P, Hartmann U. Characterization of SMOC-2, a modular extracellular calcium-binding protein. Biochem J 2003; 373(3):805–14.

31. Bradshaw AD. Diverse biological functions of the SPARC family of proteins. Int J Biochem Cell Biol 2012; 44(3):480–8.

32. Wang Y, Gu J, Du A, Zhang S, Deng M, Zhao R et al. SPARC-related modular calcium binding 1 regulates aortic valve calcification by disrupting BMPR-II/p-p38 signalling. Cardiovasc Res 2022; 118(3):913–28.

33. Váncza L, Karászi K, Péterfia B, Turiák L, Dezső K, Sebestyén A et al. SPOCK1 Promotes the Development of Hepatocellular Carcinoma. Front Oncol 2022; 12:819883.

34. Edgell C-JS, BaSalamah MA, Marr HS. Testican-1: a differentially expressed proteoglycan with protease inhibiting activities. Int Rev Cytol 2004; 236:101–22.

35. Shi J-H, Hao Y-J. DDX10 overexpression predicts worse prognosis in osteosarcoma and its deletion prohibits cell activities modulated by MAPK pathway. Biochem Biophys Res Commun 2019; 510(4):525–9.

36. Li B, Cui H, Liu W, Lan Z, Liu C, Yang Y et al. DDX10 Exacerbates Exosomal PD-L1-Dependent T Cell Exhaustion via Phase Separation of Rab27b in Oral Squamous Cell Carcinoma. Research (Wash D C) 2025; 8:697.

37. Li J, Zhou Y, Zhao W, Liu J, Ullah R, Fang P et al. Porcine reproductive and respiratory syndrome virus degrades DDX10 via SQSTM1/p62-dependent selective autophagy to antagonize its antiviral activity. Autophagy 2023; 19(8):2257–74.

38. Liu C, Tang J, Duan X, Du Y, Wang X, Cui Y. DDX10 promotes human lung carcinoma proliferation by U3 small nucleolar ribonucleoprotein IMP4. Thorac Cancer 2021; 12(12):1873–80.

39. Zhou X, Liu Z, He T, Zhang C, Jiang M, Jin Y et al. DDX10 promotes the proliferation and metastasis of colorectal cancer cells via splicing RPL35. Cancer Cell Int 2022; 22(1):58.

40. Siebold B, Deutzmann R, Kühn K. The arrangement of intra- and intermolecular disulfide bonds in the carboxyterminal, non-collagenous aggregation and cross-linking domain of basement-membrane type IV collagen. Eur J Biochem 1988; 176(3):617–24.

41. Basak T, Vega-Montoto L, Zimmerman LJ, Tabb DL, Hudson BG, Vanacore RM. Comprehensive Characterization of Glycosylation and Hydroxylation of Basement Membrane Collagen IV by High-Resolution Mass Spectrometry. J Proteome Res 2016; 15(1):245–58.

42. Colorado PC, Torre A, Kamphaus G, Maeshima Y, Hopfer H, Takahashi K et al. Anti-angiogenic cues from vascular basement membrane collagen. Cancer Res 2000; 60(9):2520–6.

43. Saito K, Chen M, Bard F, Chen S, Zhou H, Woodley D et al. TANGO1 facilitates cargo loading at endoplasmic reticulum exit sites. Cell 2009; 136(5):891–902.

44. Magnon C, Galaup A, Mullan B, Rouffiac V, Bouquet C, Bidart J-M et al. Canstatin acts on endothelial and tumor cells via mitochondrial damage initiated through interaction with alphavbeta3 and alphavbeta5 integrins. Cancer Res 2005; 65(10):4353–61.

45. Des Parkin J, San Antonio JD, Pedchenko V, Hudson B, Jensen ST, Savige J. Mapping structural landmarks, ligand binding sites, and missense mutations to the collagen IV heterotrimers predicts major functional domains, novel interactions, and variation in phenotypes in inherited diseases affecting basement membranes. Hum Mutat 2011; 32(2):127–43.

46. Kamphaus GD, Colorado PC, Panka DJ, Hopfer H, Ramchandran R, Torre A et al. Canstatin, a novel matrix-derived inhibitor of angiogenesis and tumor growth. J Biol Chem 2000; 275(2):1209–15.

47. Kuo CJ, LaMontagne KR, Garcia-Cardeña G, Ackley BD, Kalman D, Park S et al. Oligomerization-dependent regulation of motility and morphogenesis by the collagen XVIII NC1/endostatin domain. J Cell Biol 2001; 152(6):1233–46.

48. Kim Y-M, Hwang S, Kim Y-M, Pyun B-J, Kim T-Y, Lee S-T et al. Endostatin blocks vascular endothelial growth factor-mediated signaling via direct interaction with KDR/Flk-1. J Biol Chem 2002; 277(31):27872–9.

49. O'Reilly MS, Boehm T, Shing Y, Fukai N, Vasios G, Lane WS et al. Endostatin: an endogenous inhibitor of angiogenesis and tumor growth. Cell 1997; 88(2):277–85.

50. Ortega N, Werb Z. New functional roles for non-collagenous domains of basement membrane collagens. J Cell Sci 2002; 115(Pt 22):4201–14.

51. Karumanchi SA, Jha V, Ramchandran R, Karihaloo A, Tsiokas L, Chan B et al. Cell surface glypicans are low-affinity endostatin receptors. Mol Cell 2001; 7(4):811–22.

52. Rehn M, Veikkola T, Kukk-Valdre E, Nakamura H, Ilmonen M, Lombardo C et al. Interaction of endostatin with integrins implicated in angiogenesis. Proc Natl Acad Sci U S A 2001; 98(3):1024–9.

53. Rosell-García T, Paradela A, Bravo G, Dupont L, Bekhouche M, Colige A et al. Differential cleavage of lysyl oxidase by the metalloproteinases BMP1 and

ADAMTS2/14 regulates collagen binding through a tyrosine sulfate domain. J Biol Chem 2019; 294(29):11087–100.

54. Pilecki B, Holm AT, Schlosser A, Moeller JB, Wohl AP, Zuk AV et al. Characterization of Microfibrillar-associated Protein 4 (MFAP4) as a Tropoelastin- and Fibrillin-binding Protein Involved in Elastic Fiber Formation. J Biol Chem 2016; 291(3):1103–14.

55. Eisenmann KM, McCarthy JB, Simpson MA, Keely PJ, Guan JL, Tachibana K et al. Melanoma chondroitin sulphate proteoglycan regulates cell spreading through Cdc42, Ack-1 and p130cas. Nat Cell Biol 1999; 1(8):507–13.

56. Burg MA, Tillet E, Timpl R, Stallcup WB. Binding of the NG2 proteoglycan to type VI collagen and other extracellular matrix molecules. J Biol Chem 1996; 271(42):26110–6.

57. Barritt DS, Pearn MT, Zisch AH, Lee SS, Javier RT, Pasquale EB et al. The multi-PDZ domain protein MUPP1 is a cytoplasmic ligand for the membrane-spanning proteoglycan NG2. J Cell Biochem 2000; 79(2):213–24.

58. Fukushi J, Makagiansar IT, Stallcup WB. NG2 proteoglycan promotes endothelial cell motility and angiogenesis via engagement of galectin-3 and alpha3beta1 integrin. Mol Biol Cell 2004; 15(8):3580–90.

59. Goretzki L, Burg MA, Grako KA, Stallcup WB. High-affinity binding of basic fibroblast growth factor and platelet-derived growth factor-AA to the core protein of the NG2 proteoglycan. J Biol Chem 1999; 274(24):16831–7.

60. Goretzki L, Lombardo CR, Stallcup WB. Binding of the NG2 proteoglycan to kringle domains modulates the functional properties of angiostatin and plasmin(ogen). J Biol Chem 2000; 275(37):28625–33.

61. Price MA, Colvin Wanshura LE, Yang J, Carlson J, Xiang B, Li G et al. CSPG4, a potential therapeutic target, facilitates malignant progression of melanoma. Pigment Cell Melanoma Res 2011; 24(6):1148–57.

62. Erusappan P, Alam J, Lu N, Zeltz C, Gullberg D. Integrin α11 cytoplasmic tail is required for FAK activation to initiate 3D cell invasion and ERK-mediated cell proliferation. Sci Rep 2019; 9(1):15283.

63. Primac I, Maquoi E, Blacher S, Heljasvaara R, van Deun J, Smeland HY et al. Stromal integrin α11 regulates PDGFR-β signaling and promotes breast cancer progression. J Clin Invest 2019; 129(11):4609–28.

64. Pellinen T, Arjonen A, Vuoriluoto K, Kallio K, Fransen JAM, Ivaska J. Small GTPase Rab21 regulates cell adhesion and controls endosomal traffic of beta1-integrins. J Cell Biol 2006; 173(5):767–80.
